# Analysis of the Effect of Skin Pigmentation and Oxygen Saturation on Monte Carlo-Simulated Reflectance Photoplethysmography Signals

**DOI:** 10.3390/s25020372

**Published:** 2025-01-10

**Authors:** Raghda Al-Halawani, Meha Qassem, Panicos A. Kyriacou

**Affiliations:** Research Centre for Biomedical Engineering, City St George’s, University of London, London EC1V 0HB, UK; meha.qassem@city.ac.uk (M.Q.); p.kyriacou@city.ac.uk (P.A.K.)

**Keywords:** Monte Carlo simulation, photoplethysmography, skin pigmentation, oxygen saturation, pulse oximetry

## Abstract

The effect of skin pigmentation on photoplethysmography and, specifically, pulse oximetry has recently received a significant amount of attention amongst researchers, especially since the COVID-19 pandemic. With most computational studies observing overestimation of arterial oxygen saturation (SpO_2_) in individuals with darker skin, this study seeks to further investigate the root causes of these discrepancies. This study analysed intensity changes from Monte Carlo-simulated reflectance PPG signals across light, moderate, and dark skin types at oxygen saturations of 70% and 100% in MATLAB R2024a. With simulated intensity reflecting PPG amplitude, the results showed that systolic intensity decreased by 3–4% as pigmentation increased at 660 nm. It was also shown that the impact at 940 nm is minimal (<0.2%), indicating that the increased absorption of red light by melanin has a greater effect on the ratio of ratios calculations. These results suggest that in-built adjustments may be required for data collected from red-light sources in pulse oximeters that do not currently have the necessary post-processing algorithms to account for this difference between diverse skin populations.

## 1. Introduction

Photoplethysmography (PPG) is extensively utilised in medical diagnostics to optically and non-invasively monitor blood volume changes within the microvascular bed of tissue. The appeal of PPG lies in its simplicity, cost-effectiveness, and its ability to provide essential physiological metrics, such as heart rate and oxygen saturation (SpO_2_). This makes it an indispensable tool in various healthcare environments, from routine monitoring to critical care [1]. However, the accuracy and reliability of PPG measurements are influenced by various factors, including skin pigmentation, which has received considerable attention, particularly since the COVID-19 pandemic [2,3]. Specifically, research has shown that skin pigmentation can alter the way light is absorbed and scattered within biological tissue, potentially leading to inaccuracies in SpO_2_ measurements [4]. Therefore, understanding and addressing the impact of skin pigmentation on PPG is crucial for improving the technology’s effectiveness across diverse patient populations.

Several studies have explored the effect of melanin concentration, the primary determinant of skin colour, on SpO_2_ measurements using the Monte Carlo technique [5,6,7,8]. Employing this type of simulation has sought to replicate and better understand the discrepancies observed in clinical settings. These studies have consistently indicated an overestimation of SpO_2_ in subjects with darker skin, particularly evident in the shift in simulated calibration curves for both transmittance and/or reflectance pulse oximetry modes in comparison to light skin populations. While this research has been crucial for establishing the influence of red-light absorption by increasing levels of melanin, some improvements in the methodologies can help create more realistic models of the human finger which, in effect, can improve PPG data obtained for different skin types.

Consequently, this study aims to examine the effect of skin pigmentation on simulated Monte Carlo PPG signals using red light (660 nm) and infrared light (940 nm), which, to our knowledge, has not yet been explored. The aim of this research is to directly explore how skin pigmentation affects PPG signals through an in silico investigation, which provides complementary insights to the studies that have focused on simulated pulse oximeter calibrations for different skin types [5,6,7,9,10]. Whilst they have been crucial for understanding variations in the ratio of ratios values, analysing the initial signal distortions caused by skin pigmentation provides clarity to the overall knowledge base. This approach allows for observing the signal’s amplitude and quality with different skin types, offering insights that can lead to more precise adjustments in calibration algorithms before the raw PPG data are processed.

Additionally, another novelty in this study is improving the modelling of skin pigmentation. One of the key advancements is the use of spectrophotometrically obtained optical properties for different skin tones rather than relying on mathematical equations. Traditionally, the Fitzpatrick scale evaluates parameters related to predicting the skin’s reaction to ultraviolet light [11]. As a result, it may not account for all aspects of skin colour, such as undertones, redness, or colour evenness [12]. This approach risks introducing bias into the subjective stratification of skin pigmentation, which may reduce the reliability and applicability of traditional phototyping in clinical research. For instance, a study by He et al. showed that patient self-reported race and pigmentary phenotypes are inaccurate predictors of sun sensitivity, as defined by the Fitzpatrick scale [13]. Moreover, another study presented by Pershing et al. employed reflectance spectrophotometry to quantify skin pigmentation in anatomical sites that are both protected and unprotected from UV radiation [14]. With a motivation to also assess the correlation between objective skin pigmentation stratification methods and the Fitzpatrick scale, they found that, in some cases, there is a lack of complete agreement between the phenotype group assigned to the patient by the clinician and the spectra obtained. Hence, greater accuracy in the methodology approach can be achieved when objectively quantifying skin pigmentation.

## 2. Materials and Methods

Monte Carlo models simulate the path of virtual photon clusters as they travel through biological tissue with defined optical properties. During their travel, they undergo multiple instances of absorption, scattering, and reflection. Photons propagate in three-dimensional (3D) space using a Cartesian coordinate system (x, y, z) and spherical polar coordinate system, which is essential for accurately illustrating the distribution of light in all possible directions. When photons interact with scatterers in the tissue and its path changes, the resulting directional shift is first computed in the spherical polar system before being converted to the Cartesian coordinate system.

**Figure 1:**
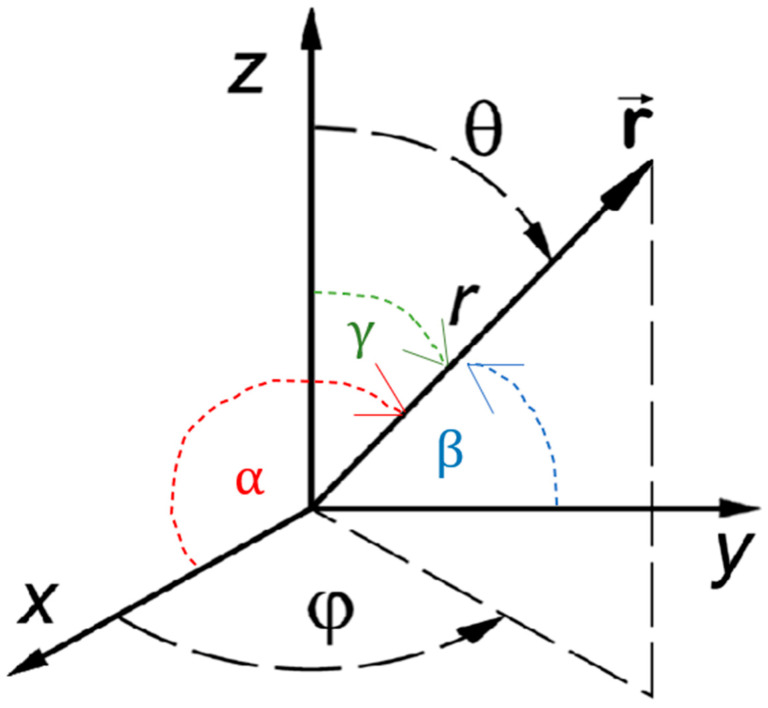
The relationship between the cartesian coordinate and spherical polar coordinate system. The vector $\vec{r}$ makes the deflection angle (θ) and the azimuthal angle (φ) in the spherical polar coordinate system. Similarly, the position vector $\vec{r}$ forms angles with α, β, and γ with the x, y, and z axes, respectively.

To represent the direction of a photon at any given point, a vector $\vec{r}$ is used. In the spherical polar coordinate system, this direction is defined by the deflection angle (θ) and the azimuthal angle (φ), as illustrated in Figure 1. In the Cartesian system, the position vector $\vec{r}$ forms angles with α, β, and γ with the x, y, and z axes, respectively. These relationships allow for the transformation between the spherical polar and Cartesian coordinate systems through direction cosines given by $u_{x}$, $u_{y}$, $u_{z}$, which are given by the following:

(1a) $$u_{x}=\cos\alpha =\sin\theta \cos\varphi$$

(1b) $$u_{y}=\cos\beta =\sin\theta \sin\varphi$$

(1c) $$u_{z}=\cos\gamma =\cos\theta$$

where φ=2πξ, a randomly generated azimuthal angle between 0 and 2π to represent a full circle. In MATLAB, the ‘rand’ function generates a random number (ξ) between 0 and 1 based on a uniform distribution, which means that each value in the range has an equal chance of being selected. It uses a deterministic algorithm, such as the Mersenne Twister, which determines the entire sequence of numbers based on an initial ‘seed’ value. If the same seed is used with the same settings, the sequence of random numbers will be identical each time, making experiments reproducible. Therefore, if the same simulation is run multiple times, it will not produce the same exact data but should generate the same trends observed between different inputs and outputs.

Other parameters that are randomly generated are the photon step size, which defines the distance that the photons travel between each scattering event, the position coordinates of the photons, and the radius of the gaussian beam. Briefly, the step size (l) and the gaussian beam radius ($r_{1}$) are derived by solving specific probability density functions given by:

(2) $$l=-\frac{\ln\left(\xi \right)}{\mu_{a}+\mu_{s}}$$

(3) $$r_{1}=b\sqrt{-ln(\xi )}$$

where b is the radius of the beam. Then, to determine the position of the incident photons, the x and y coordinates are calculated using basic trigonometric functions:

(4a) $$x=r_{1}\cos\varphi$$

(4b) $$y=r_{1}\sin\varphi$$

The Monte Carlo model in this study is designed to simulate characteristic points which, when combined together, emulate the alternating component of a PPG signal. This is achieved by incorporating volumetric changes in blood vessels, which reflect variations in blood pressure over time. Vessel diameters are varied as input parameters to mimic the pulsatile nature of blood flow, with the resulting light-intensity outputs representing the amplitude of a PPG signal. While the model does not directly simulate the full time-domain signal as a continuous waveform, it effectively captures the relationship between vascular dynamics and optical intensity changes, enabling the analysis of factors such as melanin concentration and its impact on PPG signal generation. The methodology will be described in detail in the subsequent paragraphs.

**Figure 2:**
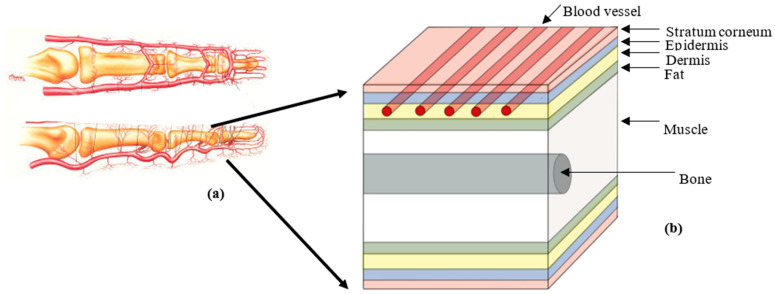
Anatomical structure of the region of interest. (**a**) Diagram of a finger showing the vasculature network to implement in the MC model [15]. (**b**) Block diagram of the finger showing the stratum corneum, epidermis, dermis and vessels, fat, muscle, and bone in alternate order.

First, a block diagram of the different layers in the human finger was created for implementation in the current Monte Carlo model (Figure 2). This included the stratum corneum, epidermis, a dermis layer containing five blood vessels positioned 1 mm apart in the lateral (x) direction, fat, and muscle containing a cylindrical bone perpendicular to the vessels, in alternate order. This way, adequate blood perfusion was modelled by including a sufficient number of blood vessels, which also covered the area where the light would travel from the source to the detector, placed 3 mm apart in the reflectance mode. The thicknesses of each layer were extracted from the literature and used in previous models, including the Monte Carlo algorithm used to simulate photons and model the different light–tissue interaction mechanisms [4,6].

Next, three silicone skin layers were prepared using different concentrations of Bismark Brown as the absorber along with a fixed concentration of titanium dioxide in Sylgard 184, specifically designed to mimic the optical and mechanical characteristics of skin. These pigmented silicone skin samples were then placed in an optical-grade cuvette and positioned inside the LAMBDA 1050+ UV/VIS/NIR spectrophotometer (PerkinElmer Inc., Waltham, MA, USA) to measure diffuse reflectance. After machine calibration, light was directed through a monochromator to the sample, and reflected light was captured by detectors for wavelengths ranging between 400 nm and 1000 nm. The diffuse reflectance spectrum was calculated by comparing the sample’s reflected light to the baseline, enabling detailed analysis of the light scattering and absorption influenced by pigmentation. After that, the spectra was processed with an Inverse Adding–Doubling (IAD) algorithm to derive the absorption and reduced scattering coefficients of the epidermal layers using an input anisotropy value of 0.8 [16]. Additionally, the L*a*b* values of the developed skin layers were calculated from the reflectance spectra, which is a colour representation system that describes colours in a three-dimensional space [17]. The optical properties of the bloodless dermis and blood vessels were calculated to reflect changes in the anatomy presented in the current model. The absorption coefficient and scattering coefficient of arterial blood ($\mu_{a\_A}$ and $\mu_{s\_A},$ respectively) and venous blood ($\mu_{a\_V}$ and $\mu_{s\_V},$ respectively) were calculated using Equations (5a)–(5d) for arterial oxygen saturation levels of 70% and 100%, as adapted from Jacques (1998) [18,19]:

(5a) $$\mu_{a\_A}={sat}_{A}*\mu_{a_{Hbo}}+\left(1-{sat}_{A}\right)*\mu_{a\_HHb}$$

(5b) $$\mu_{a\_V}={sat}_{V}*\mu_{a_{Hbo}}+\left(1-{sat}_{V}\right)*\mu_{a\_HHb}$$

(5c) $$\mu_{s\_A}={sat}_{A}*\mu_{s_{Hbo}}+\left(1-{sat}_{A}\right)*\mu_{s\_HHb}$$

(5d) $$\mu_{s\_V}={sat}_{V}*\mu_{s_{Hbo}}+\left(1-{sat}_{V}\right)*\mu_{s\_HHb}$$

${sat}_{A}$ = arterial oxygen saturation level (70% and 100%);

${sat}_{V}$ = venous oxygen saturation level (60% and 90%);

$\mu_{a_{Hbo}}$ = absorption coefficient of oxygenated blood;

$\mu_{a_{HHb}}$ = absorption coefficient of deoxygenated blood;

$\mu_{s_{Hbo}}$ = scattering coefficient of oxygenated blood;

$\mu_{s_{HHb}}$ = scattering coefficient of deoxygenated blood.

Then, the overall absorption coefficient and scattering coefficient of each vessel was calculated by taking the mean of these coefficient values to account for overall contribution from the arterial and venous blood. Lastly, the absorption coefficient of the dermal layer was calculated by taking the average of the papillary dermis, upper blood net dermis, reticular dermis, and deep blood net dermis using their respective water concentrations (Table 1):

(6) μa_dermis=μaW(n)∗vw(n)+(1−vW(n))∗7.84∗107∗λ−3.255a

$\mu_{a_{W}}$ = absorption coefficient of water;

$v_{w}$ = water concentration;

n = papillary dermis (1), upper blood net dermis (2), reticular dermis (3), and deep blood net dermis (4);

a = number of dermal sublayers (4).

The optical properties of the stratum corneum, fat, muscle, and bone layers were consistent with those also used in previous studies [4,6].

To calculate the alternating current (AC), direct current (DC) components of the simulated PPG waveforms, the perfusion index (PI), and the ratio of ratios (R) at the operating wavelengths, the systolic and diastolic intensities ($I_{s},\;I_{d}$) were utilised [4,6,19]:

(7a) $$\mathrm{AC}=I_{d}-I_{s}$$

(7b) $$\mathrm{DC}=I_{s}$$

(7c) PI=AC/DC

(7d) R=(PI)660(PI)940

**Figure 3:**
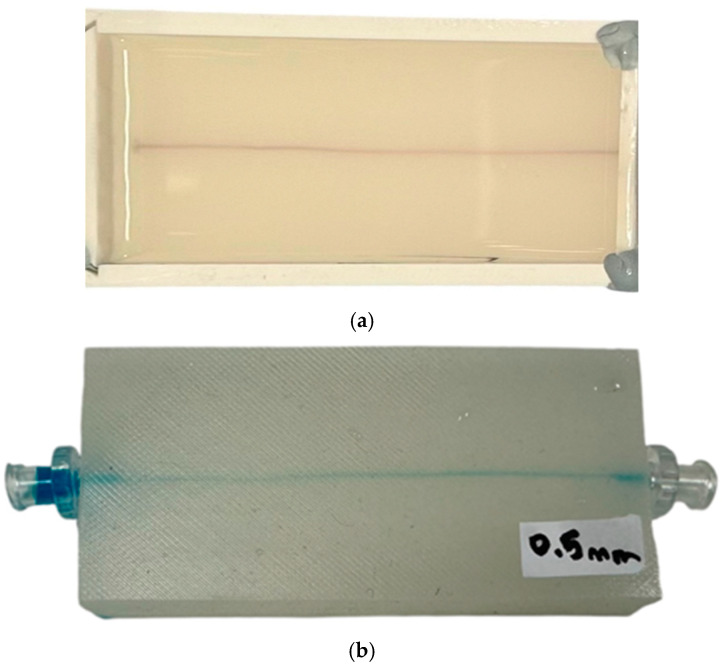
The phantom development process. (**a**) A secured 40-by-77 mm mould with a 0.5 mm diameter wire inserted through the holes on each side to create the vessel channel. Silicon is poured into the mould and left to cure for 24 h. (**b**) The cured phantom with attached connectors; the connectors supply fluid to the vessel channel from the syringe incrementally to induce pressure. Blue ink is used to visualise the vessel channel.

To simulate the characteristics of the AC component of the PPG signal, it was important to understand how the vessel volume changes when inducing different levels of internal pressure. Therefore, a custom-built phantom body with a 0.5 mm vessel channel was created by mixing 20 g of Part A Platsil Gel 00 (Polytek Development Corp, Easton, PA, USA) with 0.6 g of retarder (1.5% of the total silicone used) to extend the working time from 6 min to 10–12 min. This allowed for sufficient time to combine all components before the silicon started to harden. After that, 20 g of Part B Platsil Gel 00 was incorporated into the mixture for approximately one minute before placing it in a vacuum to remove any air bubbles. A 3D-printed 40-by-77 mm mould (width by length) was used to insert a 0.5 mm diameter wire through pre-made holes. The wire was carefully straightened and secured with weighted objects at both ends to ensure the channel remained as straight as possible. The silicone mixture was then poured into the mould and left to cure for 24 h. Once cured, the phantom was removed from the mould and the wire was extracted by pulling it to expose the channel. Figure 3 shows the vessel channel highlighted with blue ink for enhanced visualisation.

**Figure 4:**
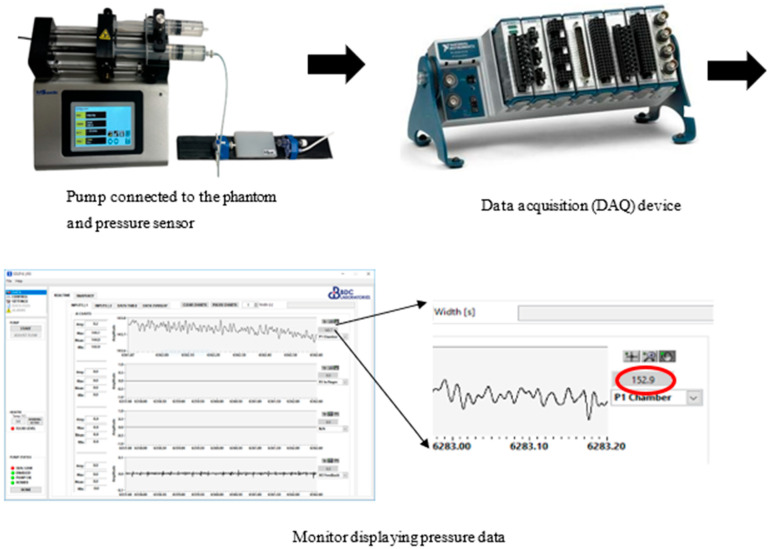
The phantom set up for a pressure–volume experiment. A pump (Legato 180, KD Scientific Inc., MA, USA) has a syringe mechanism containing blue ink to inject into the phantom. The other end of the phantom is connected to a pressure sensor to measure pressure in the vessel channel as 20 μL of blue ink is injected each time. The pressure sensor is connected to a data acquisition device (CompactDAQ–9178, National Instruments Corp., Austin, TX, USA) to process the data and display pressure readings on the monitor.

Subsequently, the phantom was connected to a pump (Legato 180, KD Scientific Inc., Holliston, MA, USA) via small connectors and attached to a syringe containing blue ink, which was injected into the channel in 20-microliter increments. This was considered a suitable increment to find a balance between reliable data collection, time, and system stability. Initially, one end of the channel was left open until it was filled with blue ink to establish a baseline volume at 0 mmHg pressure. The channel was then sealed with the other connector and an additional 20-microliter of blue ink was injected into the closed system. Each increase in volume corresponded to a specific pressure value displayed on a screen, which was recorded from the pressure sensor after 10 s from when fluid was injected into the vessel channel (Figure 4). This procedure was repeated twice for a total of seven readings, and the average pressure was plotted against the channel diameter. The diameter was calculated using the following equation:

(8a) $$V=\pi r^{2}L$$

Which was then rearranged to:

(8b) r=VπL

V = the volume of vessel channel;

r = the radius of the vessel channel;

L = the length of the vessel channel.

After that, the diameter was calculated by doubling the value of each radius.

## 3. Results

### 3.1. Optical Properties

**Figure 5:**
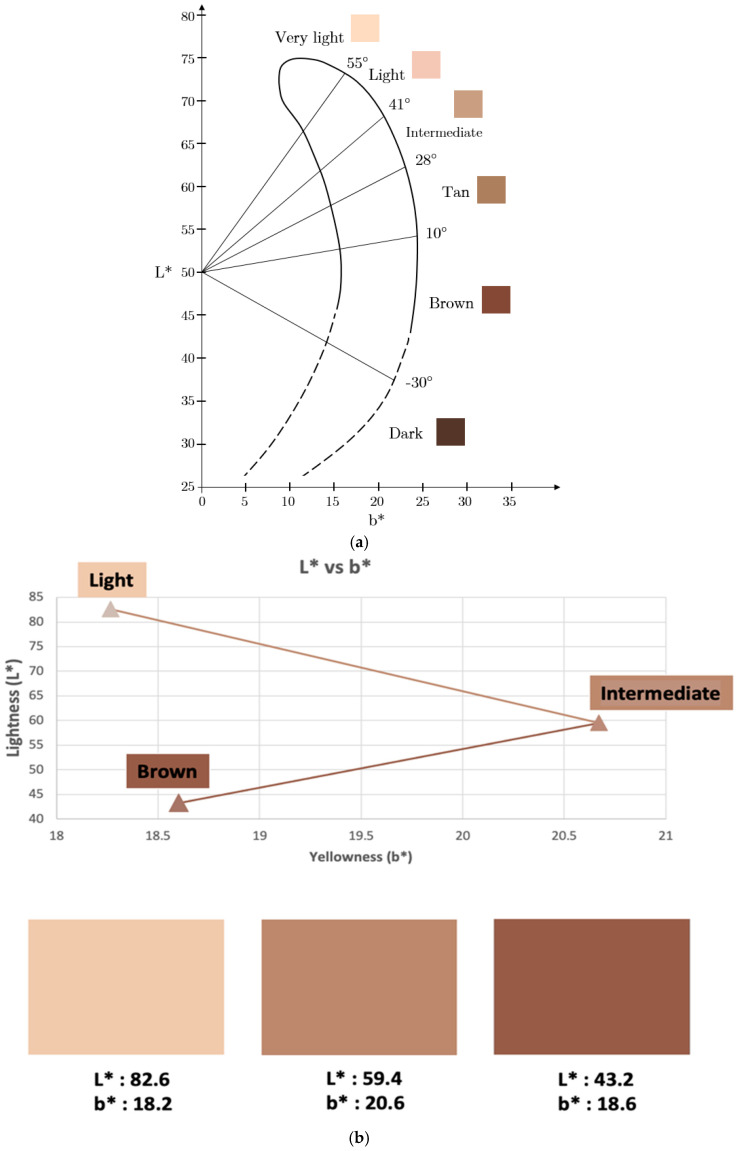
Quantification of pigmented silicon skin layers against the L*a*b* scale. (**a**) L*b* plane values for six skin types ranging between very light and dark. (**b**) L* and b* values calculated from the reflectance spectra of the developed skin layers in the Research Centre for Biomedical Engineering at City St George’s, University of London, using Microsoft Excel. The results show a similar trend to the L* and b* values reported in the literature for light, intermediate, and brown skin tones.

The L* and b* values of the silicone pigmented skin layers, corresponding to the lightness of the colour and the colour’s position between blue and yellow, respectively, are presented in Figure 5. As observed, there is a similar trend to the L* and b* values reported in the literature for light, intermediate, and brown skin tones corresponding to light, moderate, and dark skin, respectively. After validating the pigmented skin layers against the L*a*b* scale [23], the optical properties were calculated and incorporated into the model. These are presented in Table 2 along with the optical properties of the stratum corneum, fat, muscle, and bone from the literature.

### 3.2. Mechanical Properties

Changes in pressure were recorded from the pressure transducer as more and more ink was injected into the vessel channel. These values were recorded and then plotted against their respective calculated vessel diameter in MATLAB. Since there was a clear linear relationship between the two variables, a linear equation was extracted using in -built MATLAB tools for the maximum possible pressure that the system was able to withstand. This accurately represented pressure variations with vessel volume before the system was no longer functioning in a closed loop:

(9) Pressure=576∗diameter−237

**Figure 6:**
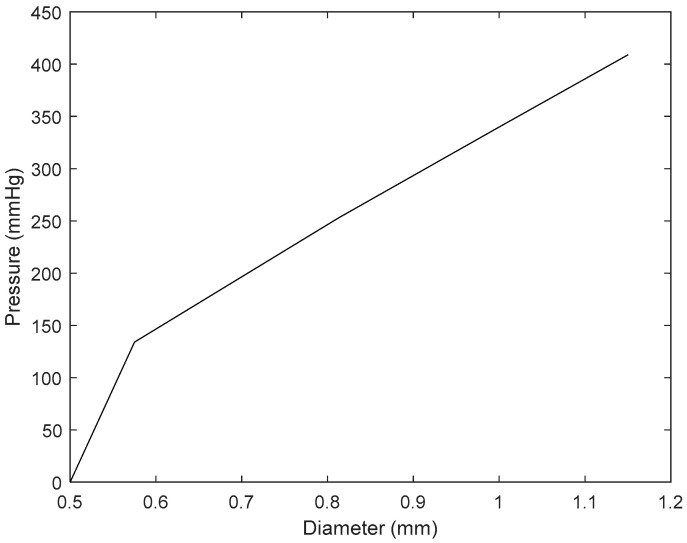
The relationship between pressure and vessel diameter in a closed-loop system. This was used to derive a linear equation to calculate variations in vessel diameter as pressure changes.

**Figure 7:**
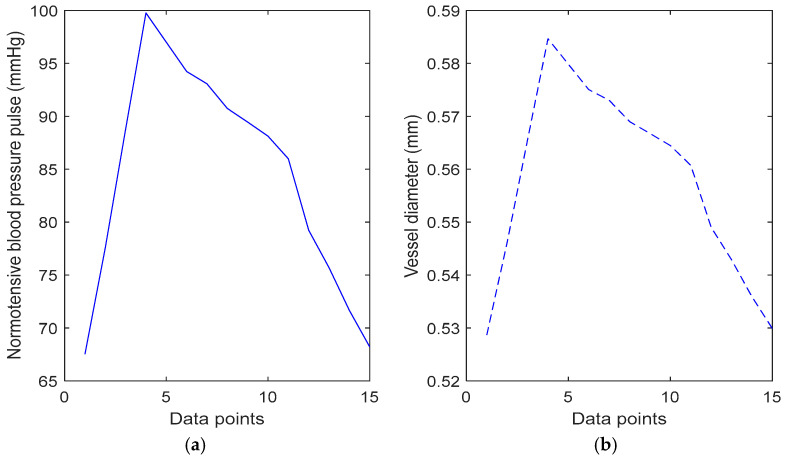
Pressure pulse used to convert volumetric blood changes into vessel diameter data using Equation (9). These 15 vessel diameter values are inputted into the Monte Carlo model to simulate characteristic points of a PPG waveform in synchrony with pressure. (**a**) Normotensive blood pressure waveform; (**b**) calculated vessel diameter values.

This equation is rearranged and used to convert variations in a normotensive blood pressure pulse (Figure 6) to changes in vessel diameter (Figure 7). The pressure pulse consists of approximately 7000 data points, which corresponds to 7000 vessel diameter values. However, simulating all these points would be computationally inefficient, so 15 key points were selected to represent the features of a PPG signal. First, the onsets of the pressure signal were identified, followed by key inflexion points, including the systolic peak and points before and after the dicrotic notch. Additional points were then chosen between these key locations to accurately capture the overall shape of the PPG signal. This vessel diameter data were input into the Monte Carlo model to simulate the dynamic response of a PPG signal.

### 3.3. Simulation Outcomes

**Figure 8:**
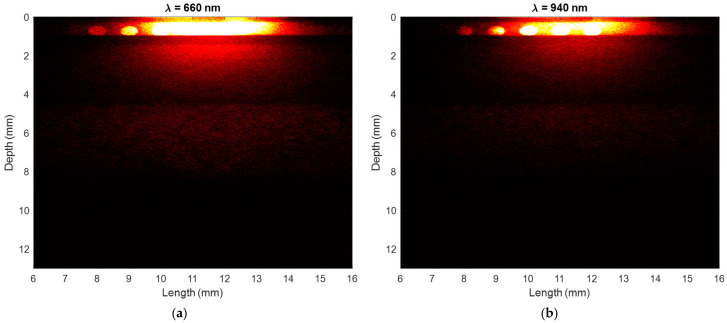
Photon profiles showing the scattering density of photons as they travel from the source to the detector. Brighter regions show a higher scattering density and vice versa. (**a**) Red light. (**b**) Infrared light.

The photon profiles, which illustrate the trajectories of photons as they travel between the source and detector, are presented in Figure 8. To plot these images, the positions of the photons were tracked along the x, y, and z directions until they were detected. The coordinates were then stored in separate vectors and organised into histograms in MATLAB to visualise the photon scattering density, with brighter red regions indicating areas of higher density. At both wavelengths, the scattering density is notably high within the vessels, particularly at 660 nm, which aligns with the magnitude of blood’s scattering coefficients (Table 2). Although photon penetration depth is quite limited to the skin layers in reflectance mode, the photons are seen to travel across a sufficient width of the finger and through the vessels. This interaction is essential for generating reproducible characteristic points where the key features of the PPG signal are visibly clear and well distinguished. The high scattering coefficient facilitates photon transport and interaction with the blood in the vessels, which enhances the signal-to-noise ratio by increasing the light modulation corresponding to pulsatile blood flow. However, it should be noted that the scattering coefficient alone does not directly minimise noise but rather supports conditions conducive to a higher-quality PPG signal.

**Figure 9:**
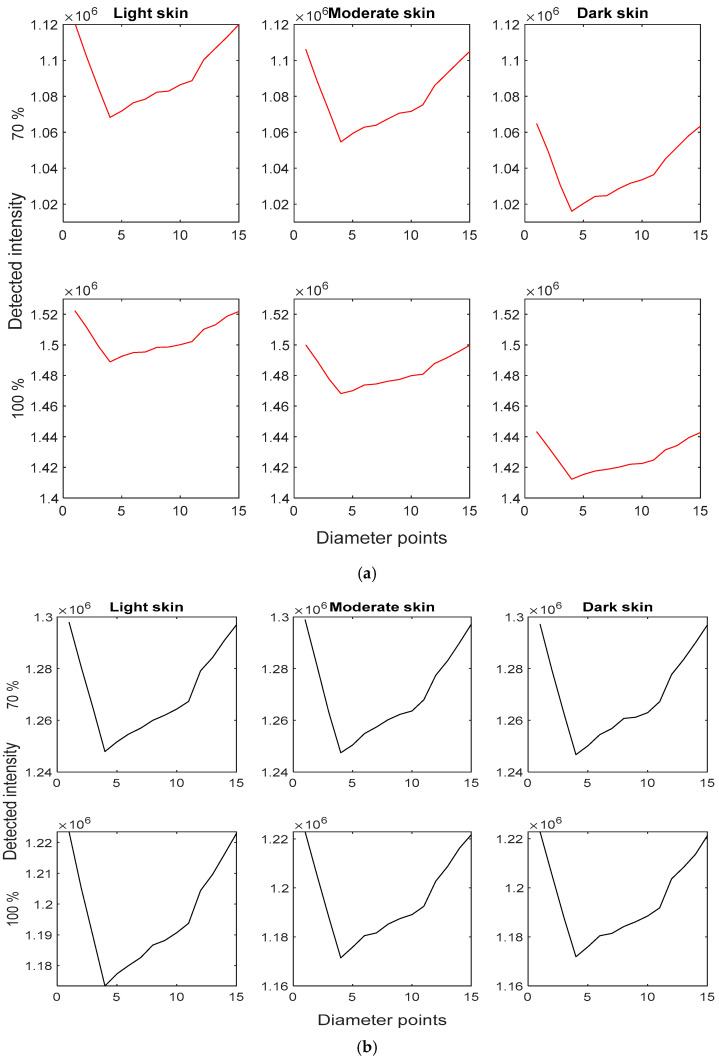
Simulated photoplethysmography signals from the Monte Carlo model for light, moderate and dark skin at 70% and 100% oxygen saturation levels. (**a**) Red light. (**b**) Infrared light.

By employing the methodology outlined in this study, PPG characteristic points were successfully simulated from the Monte Carlo model, clearly illustrating the systolic and diastolic slopes, systolic peaks, onsets, and dicrotic notches. These signals were generated for light, moderate, and dark skin at 70% and 100% oxygen saturation with red and infrared light, as shown in Figure 9.

Notably, at 660 nm, the detected intensity decreases as skin pigmentation becomes darker. Additionally, the intensity at 100% saturation is greater than at 70%, since deoxygenated haemoglobin absorbs more light at this wavelength compared to oxygenated haemoglobin, resulting in reduced detected intensity at lower oxygen levels. In contrast, with infrared light, higher intensities are observed at 70% oxygen saturation since deoxygenated haemoglobin absorbs less light. However, changes in detected intensity are minimal as skin pigmentation increases, suggesting that this absorber has a lesser effect on PPG amplitude compared to red light. This trend is evident in the percentage difference in systolic intensities between light, moderate, and dark skin, as shown in Table 3. For red light, the percentage difference between light and moderate skin exceeds 1% and rises to over 4–5% between light and dark skin. However, even for dark skin, the maximum percentage difference for infrared light is just above 0.1%, implying that the impact on the systolic peak with infrared light is approximately 50 times less than red light. Furthermore, changes in oxygen saturation result in a 0.11% and 0.27% difference between light and moderate skin and light and dark skin, respectively, at 660 nm, and 0.13% and 0.031% at 940 nm. These findings once again confirm the trends in haemoglobin absorption spectra at the two wavelengths consistently across different skin pigmentations.

**Figure 10:**
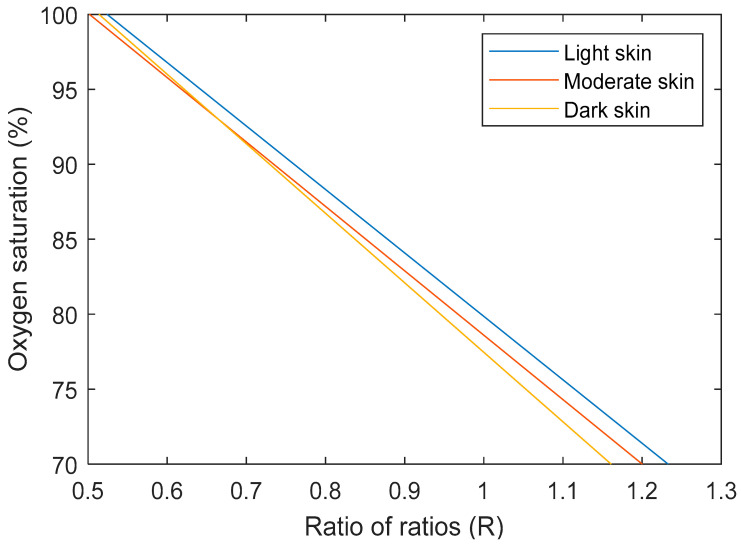
Simulated ratios of ratios plotted against arterial oxygen saturation for light, moderate, and dark skin.

Lastly, further analysis examined the calculated perfusion index values, which represent the ratio between the AC and direct current (DC) components of the simulated PPG signals for red and infrared light at 70% and 100% oxygen saturation for light, moderate, and dark skin (Table 4). At 70% oxygen saturation, the perfusion index decreases with red light as skin pigmentation becomes darker; it then increases with infrared light. Consequently, the overall ratio of ratios (R), calculated by taking the ratio between the red and infrared perfusion indices (Equation (7d)), decreases as pigmentation increases, as shown in Figure 10. However, at 100% oxygen saturation, there is no direct relationship between the perfusion index and skin type. This can be attributed to the differences in the optical properties of blood when it is more oxygenated in addition to the non-linearity of the scattering coefficients of the epidermal layers (Table 2). This increase in scattering may result in it being the dominant mechanism of the light–tissue interactions taking place and hence increasing the random redirection of the photons. Despite this, the importance of analysing the ratio of ratios instead of solely examining intensity changes or perfusion indices at individual wavelengths is highlighted. The ratio method captures the interplay between contributions from different tissue components at both wavelengths, showing that skin pigmentation has a minimal impact on pulse oximeter calibration at 100% oxygen saturation compared to 70%. While there are a number of studies relating the perfusion index to several biomarkers, including oxygen, lactate, glucose, etc., a potential area of research could involve the assessment of the perfusion index at specific wavelengths of interest. This would serve as a useful validation tool to compare with such data derived from computational models.

## 4. Discussion

This study presents a comprehensive Monte Carlo model of the human finger to examine the effects of skin pigmentation on simulated characteristic points of PPG signals at two oxygen saturation levels. The model was successfully implemented, and the analysis of the results from the simulations align with findings in some of the existing literature regarding the overestimation of SpO_2_ in patients with darker skin pigmentation, especially at lower saturation levels [24,25,26,27].

A key innovation of this work lies in its methodological improvements for characterising the optical properties of the skin layers, producing a more realistic finger model. The pigmented skin layers were validated against the L*a*b* scale, which is a more objective method for stratifying skin pigmentation in comparison to the Fitzpatrick scale. As a result, this ensured that the optical properties incorporated into the current Monte Carlo model more accurately reflected real-world variations in skin pigmentation. This is an advancement from previous studies [6,8] where the optical properties of the epidermis were calculated using equations from the literature [18] for skin types based on a subjective scale. Although variations in the perfusion index at 940 nm were consistent with previous studies [6], which further confirms the minimal influence of melanin concentration at this wavelength, the perfusion index at 660 nm was significantly more affected. This difference can be attributed to the greater magnitude in absorption coefficients (0.73 ${mm}^{-1}$, 4.21 ${mm}^{-1}$, and 8.24 ${mm}^{-1}$ for light, moderate, and dark skin, respectively), which impact light–tissue interactions and intensity predictions in the model. These findings suggest that varying the melanin concentration alone may be an insufficient approach to accurately quantifying epidermal absorption coefficients and highlights the complexity of optically characterising skin colour beyond simple equations. Overall, this characterisation was essential for generating more representative magnitudes of simulated detected intensities which better reflect the effect of light absorption and scattering with different skin types. Furthermore, the elastic properties of the vessel were characterised through the development of a custom phantom, which enabled an analysis of the relationship between the vessel channel diameter and internal pressure based on a derived equation. However, as improvements in obtaining the optical properties of the skin layers have been shown, the same effort should be applied to capturing the complex mechanical properties of blood vessels to improve the modelling of peripheral haemodynamics in future work. One key area to focus on is the active regulation in the vessels, such as vasodilation and vasoconstriction, which control how vessels expand and contract in response to blood flow and pressure changes. These dynamic processes are important for simulating real-world conditions like stress, exercise, or disease. Additionally, the non-linear stiffness of vessel walls, which behave differently under varying pressures and include more realistic blood flow dynamics and viscosity, should be considered.

Moreover, the photon profiles in Figure 8 revealed that scattering density is notably higher in blood vessels, particularly at 660 nm. This observation aligns with the known scattering properties of blood and indicates that the simulated finger model effectively captures the interaction between the photons and the tissue layers. Despite high scattering densities, photon penetration was limited to the skin layers in reflectance mode, a phenomena also observed in previous studies [4,8,19]. The PPG signals displayed consistent features across different skin types and oxygen saturation levels, and they suggested that skin pigmentation effects detected intensity, which is indicative of amplitudes from experimental-based PPG signals. More analysis on the simulated amplitude of the PPG signal at 660 nm is necessary, as higher levels of pigmentation showed a greater affect at this wavelength in comparison to the infrared PPG signals. The results confirm that red light is more sensitive to changes in skin pigmentation than infrared light, with percentage differences in systolic intensities being significantly higher for red light (Table 3). This differential impact is attributed to the varying absorption of deoxygenated and oxygenated haemoglobin at different wavelengths. However, further analysis of the perfusion index is vital when accounting for changes in oxygen saturation as well as skin pigmentation. Overall, the findings highlight the importance of accounting for skin pigmentation in pulse oximeter calibration at different oxygen saturation levels. Whilst a number of signal processing techniques have been introduced in pulse oximeter technology to minimise the effects of other known limitations, including motion artefact reduction [28,29,30], baseline drift correction [31], etc., the same is proposed to account for differences in diverse populations. This could include the integration of classification algorithms to estimate skin colour, apply pre-defined correction factors tailored to pigmentation levels, or consider scaling the absorption differences due to differences in melanin, particularly for the red-light source.

## 5. Conclusions

This study presents a detailed Monte Carlo model of the human finger, demonstrating the effects of skin pigmentation on simulated PPG signals at varying oxygen saturation levels. By incorporating the spectrophotometry measurements of developed pigmented skin layers, the model improves upon previous methodologies, offering more accurate input parameters. The results highlight how skin pigmentation alters signal intensity, particularly at 660 nm, and how the perfusion index varies between skin types at different oxygen saturation levels. These findings underscore the importance of considering diverse patient populations in device calibration or integrating post-processing algorithms to adjust for variations, promoting inclusivity in healthcare technology.

While the model successfully simulates characteristic points of PPG signals and provides valuable insights into blood pressure dynamics, certain limitations remain. The focus on volumetric vessel changes does not account for active regulatory mechanisms, such as vasodilation and vasoconstriction, which could influence PPG formation under more complex scenarios. Such considerations are important in future work to provide a more holistic representation of finger haemodynamics. Nonetheless, this work serves as a foundational framework for simulating PPG signals and understanding the effect of skin pigmentation on PPG outcomes, as well as refining peripheral haemodynamic modelling to enhance the physiological accuracy and applicability of Monte Carlo-based simulations.

## Figures and Tables

**Table 1:** Optical properties and water concentrations of different tissue constituents from the literature to calculate the absorption coefficients and scattering coefficients of arterial and venous blood, as well as the absorption coefficient of the dermis. The anisotropy factor, g, is also included to define the directionality of the photons. All values are rounded to three significant figures.

	660 nm			940 nm		
	μ_a_	μ_s_	g	μ_a_	μ_s_	g
Oxygenated haemoglobin [20]	0.150	92.3	0.985	0.650	56.8	0.977
Deoxygenated haemoglobin [20]	1.64	81.5	0.986	0.430	49.7	0.978
Water [21]	0.000400	-	-	0.0267	-	-
	Water concentration (%) [22]					
Papillary dermis	50					
Upper blood net dermis	60					
Reticular dermis	70					
Deep blood net dermis	70					

**Table 2:** Optical properties of the finger layers, including the blood vessels, at 70% and 100% oxygen saturation [6].

Tissue Layer/Component	μ_a_ (mm^−1^)		μ_s_ (mm^−1^)		g	
	660 nm	940 nm	660 nm	940 nm	660 nm	940 nm
Stratum corneum	0.0495	0.0170	25.6	5.68	0.910	0.940
Light epidermis	0.00964	0.00571	13.8	7.79	0.800	0.800
Moderate epidermis	0.0195	0.00567	12.3	7.10	0.800	0.800
Dark epidermis	0.0396	0.00627	12.9	7.70	0.800	0.800
Dermis (Bloodless)	0.0135	0.0209	25.6	5.68	0.910	0.940
Blood vessels (O_2_ = 70%)	0.672	0.573	60.6	37.1	0.985	0.977
Blood vessels (O_2_ = 100%)	0.225	0.639	87.8	54.1	0.985	0.977
Fat	0.0104	0.0170	6.20	5.42	0.900	0.900
Muscle	0.0816	0.0401	8.61	5.81	0.880	0.910
Bone	0.0351	0.0457	34.5	24.7	0.920	0.930

**Table 3:** The percentage difference in systolic intensities between light skin (reference) and moderate skin and light skin and dark skin at 660 nm and 940 nm for 70% and 100% oxygen saturation. All values are rounded to three significant figures.

	Red		Infrared	
	70%	100%	70%	100%
Light–Moderate skin	1.28	1.39	0.0356	0.166
Light–Dark skin	4.89	5.16	0.0966	0.128

**Table 4:** Simulated perfusion index (AC/DC) values for light, moderate, and dark skin at 70% and 100% oxygen saturation with 660 nm and 940 nm light sources. L = light skin, M = moderate skin, and D = dark skin.

SaO_2_ (%)	Red (660 nm)			Infrared (940 nm)		
	L	M	D	L	M	D
70	0.0484	0.0478	0.0467	0.0393	0.0398	0.0403
100	0.0221	0.0215	0.0217	0.0422	0.0429	0.0421

## Data Availability

The code and some of the data that support the findings of this article are not publicly available due to privacy. Other data not included in this article can be requested from the author at raghda.al-halawani@city.ac.uk.
